# A biocomposite-based rapid sampling assay for circulating cell-free DNA in liquid biopsy samples from human cancers

**DOI:** 10.1038/s41598-020-72163-8

**Published:** 2020-09-10

**Authors:** Bonhan Koo, Eunsung Jun, Huifang Liu, Eo Jin Kim, Yun-Yong Park, Seok-Byung Lim, Song Cheol Kim, Yong Shin

**Affiliations:** 1 Department of Convergence Medicine, Asan Medical Center, Asan Medical Institute of Convergence Science and Technology, University of Ulsan College of Medicine, Seoul, Republic of Korea; 2 Biomedical Engineering Research Center, Asan Medical Center, Asan Institute of Life Science, Seoul, Republic of Korea; 3 Division of Colon and Rectal Surgery, Department of Surgery, Asan Medical Center, University of Ulsan College of Medicine, Seoul, Republic of Korea; 4 Division of Hepatobiliary and Pancreatic Surgery, Department of Surgery, Asan Medical Center, Asan Medical Institute of Convergence Science and Technology, University of Ulsan College of Medicine, Seoul, Republic of Korea

**Keywords:** Biotechnology, Cancer

## Abstract

Cell-free nucleic acids (cfNAs) in liquid biopsy samples are emerging as important biomarkers for cancer diagnosis and monitoring, and for predicting treatment outcomes. Many cfNA isolation methods have been developed recently. However, most of these techniques are time-consuming, complex, require large equipment, and yield low-purity cfNAs because the genetic background of normal cells is amplified during cell lysis, which limits their clinical application. Here, we report a rapid and simple cfNA sampling platform that can overcome the limitations of conventional methods. We synthesised a biocomposite by combining amine-modified diatomaceous earth (DE) and cucurbituril (CB). The biocomposite platform showed high capture efficiency (86.78–90.26%) with genomic DNA and amplified DNA products (777, 525 and 150 bp). The biocomposite platform allowed the isolation of high purity and quantity cfDNAs from the plasma of 13 cancer patients (three colorectal cancer and ten pancreatic cancer samples) without requiring a lysis step or special equipment. The biocomposite platform may be useful to isolate cfNAs for the diagnosis and treatment of cancers in clinical applications.

## Introduction

The diagnosis and monitoring of cancer using liquid biopsy samples is an emerging field in cancer diagnostics because it can overcome the limitations of conventional tissue biopsy sampling, including its invasive nature, risks, and difficult reproducibility^1–3^. Liquid biopsy samples also provide important information on genomic mutations, tumour burden, and drug resistance^1–4^. Among several biomarkers in liquid biopsy samples, cell-free nucleic acids (cfNAs), which were first described in 1948, are released to the blood or other body fluids, and are important biomarkers for clinical diagnosis^5–7^. Circulating tumour DNAs (ctDNAs) are small DNA fragments derived from tumour tissues and tumour necrosis, which yield DNAs of varying sizes in contrast to uniform DNAs resulting from apoptosis of normal cells^8–10^. Recent studies demonstrated the potential of cfNAs in precision oncology for the early detection of cancer and for predicting treatment outcomes^11^.

Cell-free DNA (cfDNA) exists at low concentrations of approximately 3–22 ng per 1 mL in plasma. The ratio of ctDNA to cfDNA varies from extremely low (< 0.01%) to high (60%), according to tumour type and stage^12–14^. Although the concentration of cfDNA is higher in cancer patients than in healthy controls, the development of efficient isolation techniques to obtain high quality cfDNA in sufficient quantities is important^15–17^. In addition, contamination of gDNA derived from normal cells in the plasma can occur upon isolation of cfDNA, and reduces the sensitivity and specificity of ctDNA detection; therefore, a method for obtaining high yield and purity cfDNA is essential^18^. Novel cfDNA isolation techniques based on spin columns and magnet beads were recently developed^19–21^. However, these methods require special equipment such as a centrifuge, vacuum pump, and thermo-regulator. Moreover, the use of cell lysis buffer, which increases the yield of nucleic acids, can amplify the genetic background of noncancerous cells. To reduce the genetic background while increasing the detection sensitivity of ctDNA obtained from liquid biopsy, Jin et al*.* developed a simple microfluidic platform to isolate cfDNA from colorectal cancer (CRC) samples without cell lysis^22^. The development of novel techniques for isolating cfNA from liquid biopsy samples would be desirable in several cancers.

Pancreatic cancer is a severe and lethal malignancy, and the poor prognosis of patients with pancreatic cancer is mainly attributed to its frequent diagnosis at advanced stages^23–25^. The clinical use of cfDNA as a biomarker for early detection and monitoring of metastasis and treatment outcomes may improve the survival rate and prognosis of cancers, including pancreatic cancer^25^. Recent studies show a high correlation between cfDNA level and cancer stage, supporting the value of cfDNA as a biomarker for the diagnosis and monitoring of human malignancies^26,27^. However, there is no standard method of cfNA isolation and analysis for clinical application.

In this study, we report a simple, low-cost cfNA isolation platform for the rapid and accurate diagnosis and monitoring of cancer patients using patient-derived plasma. This platform is based on the use of a novel biocomposite of diatomaceous earth (DE) and cucurbit[6]uril (CB). DE consists of porous silica microparticles of 1–200 µm^28^. CB is composed of n-glycoluril units, negatively charged carbonyl groups and a cavity^29–32^. We synthesised the biocomposites by coating the amine groups on the surface of DE with CB. This biocomposite platform allowed the effective isolation of DNA when genomic DNA (> 1 kb) and amplified products (777, 525 and 150 bp) were used. The clinical utility of the biocomposite platform for cfDNA isolation from plasma was validated in three patients with CRC and ten patients with pancreatic cancer. The biocomposite platform allowed rapid (< 20 min) isolation of cfDNA from clinical specimens. The results suggest that the biocomposite platform can be used for cfNA isolation from liquid biopsy specimens for various clinical applications.

## Methods

### Fabrication and operation of the biocomposite platform

For cfNA sampling using DE (D3877, Sigma-Aldrich, St. Louis, MO, USA), amine-modified diatomaceous earth (AD) was fabricated in three steps as follows. (1) DE washing step: 3 g DE was added to 150 mL ultrapure distilled water (DW) while stirring at 550 rpm for 10 min at room temperature (RT). The stirring was stopped for 1 min, and the supernatant was discarded. The precipitate was then washed twice with 150 mL DW under the same conditions. After washing, the mixture was divided into 50 mL conical tubes, centrifuged at 14,000 rpm for 1 min, the supernatant was discarded, and the precipitate was stored. (2) 3-aminopropyl-methyl-diethoxysilane (APDMS, 371890, Sigma-Aldrich, St. Louis, MO, USA) functionalisation step: 3 mL APDMS was added to 150 mL ethanol-DW (95:5, v/v) solution while stirring at 550 rpm for 3 min at RT. The pure DE precipitate was mixed with a 95% ethanol solution with APDMS and stirred at 550 rpm for 4 h at RT. The precipitate was washed three times with 95% ethanol to remove the remaining APDMS. After washing, the mixture was divided into 50 mL conical tubes and centrifuged at 14,000 rpm, and the supernatant was discarded. (3) *Dry step* the pure AD was dried for 2 days at RT in a vacuum chamber until all remaining ethanol was evaporated. The biocomposite for cfDNA sampling was prepared as follows: 50 mg AD was added into a 1 mL of CB (94544-1G-F, Sigma-Aldrich, St. Louis, MO, USA) solution containing 0.1, 0.01, or 0.001 mg CB, and incubated at RT for 2, 4, or 6 h to make biocomposite with shaking for 1 min at 30 min intervals. 1 mL of the biocomposite mixture containing 50 mg of AD and 0.001 mg CB was prepared for cfDNA isolation. For the capture of amplified DNA and cfDNA, 40 μL of the prepared biocomposite was added to 500 μL blood plasma or 500 μL solution containing amplified DNA, and then incubated at RT for 10 min with shaking for 30 s at 2 min intervals. After the capture, the supernatant was discarded by centrifugation at 5,500 rpm for 1 min, and the precipitate was washed by pipetting with the addition of 1 mL PBS. Then, the precipitate was washed two more times with 1 mL PBS under the same conditions. Finally, 100 μL elution buffer (pH 10.6) was added to collect the captured amplified DNA or cfDNA for further analysis. The zeta potential of the DE, AD, and biocomposite was measured by dynamic light scattering (DynaPro NanoStar, Wyatt, GA, USA). Fourier transform infrared spectroscopy (FTIR) results and scanning electron microscope (SEM) images of DE, AD, biocomposite, and biocomposite-cfDNA were obtained from FT-IR Fourier Transform Infrared Spectrometer (TENSOR27, Bruker, Germany) and field emission scanning electron microscope (JSM-7800F Prime, JEOL Ltd, Japan).

### Conventional PCR assay

Conventional assays, such as end-point PCR and real-time PCR, were used with the biocomposite platform to test its utility. The forward and reverse primers were synthesised at the usual length of 24 bp (Table S1). A Taq PCR Core Kit (201225, Qiagen, Hilden, Germany) was used to produce amplified 777, 525, and 150 bp DNAs for further use. The end-point PCR process consisted of an initial denaturation step at 95 °C for 15 min; 40 cycles of 95 °C for 30 s, 57–62 °C for 30 s, and 72 °C for 30 s; and a final elongation step at 72 °C for 10 min. A volume of 5 μL DNA was amplified in a total volume of 25 μL containing 10 × PCR buffer, 2.5 mM MgCl, 0.25 mM deoxynucleotide triphosphate (dNTP), 25 pmol of each primer, and 1 unit of Taq DNA polymerase. Gel electrophoresis was performed to separate PCR products on a 2% agarose gel containing LoadingSTAR (A750, Dyne Bio Inc., Seoul, Korea). The gel was visualised using a ChemiDoc XRS + system (Bio-Rad, Marnes-la-Coquette, France). Brilliant III SYBR Green QPCR Master Mix (600882, Agilent Technologies, Wilmington, DE, USA) was used to produce a standard curve in which the template concentration was unknown, and cfDNA integrity (*Alu* 247/115 ratio) and cellular DNA background (β-actin 400 bp) were measured. The real-time PCR procedure used was modified from the Bio-Rad CFX96 Instrument protocol. Briefly, 5 μL DNA was amplified in a total volume of 20 μL containing 2 × brilliant III SYBR Green QPCR Master Mix and 25 pmol of each primer. For *Alu* elements, the amplification protocol consisted of an initial denaturation step at 95 °C for 10 min, 35 cycles of 30 s at 95 °C, 30 s at 62 °C, 30 s at 72 °C, and a cooling step at 40 °C for 30 s. The absolute equivalent amount of cfDNA in each sample was determined using a calibration curve with serial dilutions of genomic DNA obtained from healthy donors. For others, the amplification protocol consisted of an initial denaturation step at 95 °C for 15 min, 40–45 cycles of 10 s at 95 °C, 20 s at 57–62 °C, and 20 s at 72 °C, and a cooling step at 40 °C for 30 s. The amplified products with SYBR Green signals were obtained using a CFX96 Real-Time PCR System (Bio-Rad, Marnes-la-Coquette, France).

### Evaluation of the biocomposite platform

To evaluate the capture efficiency of the biocomposite platform, gDNA was extracted from HCT116 human CRC cells (ATCC_CCL-247), and PCR products of 777, 525, and 150 bp were generated. The gDNA of HCT116 cells was extracted using the QIAamp DNA Mini Kit (51306, Qiagen, Hilden, Germany) and stored at − 20 °C until use. The amplified PCR products were generated using clinical specimens derived from patients as described in previous studies^22,33–35^. The products of 777 and 150 bp consisted of part of exon 2 containing the *KRAS gene* sequence and were used as a template for nucleic acid amplification using a *KRAS* 133 and 150 bp primers. The product of 525 bp consisted of part of the *C. burnetii transposase gene* containing the *IS1111a* region and was used as a template for nucleic acid amplification using a 203 bp primer. All PCR products were stored at − 20 °C until use.

### Cell-free DNA extraction from clinical specimens

To validate the biocomposite platform for clinical use, blood plasma specimens from three CRC and ten pancreatic cancer patients were collected from the Bio Resource Center (BRC) of the Asan Medical Center (Seoul, Korea, No. 2016-13(125) & 2019-8(187)) after approval from the Institutional Review Board (IRB_ 2016-0809 & IRB_ 2019-0631). A statement to confirm that all methods were carried out in accordance with relevant guidelines and regulations. The Institutional Review Board of Asan Medical Center approved the study protocol, and informed consent was obtained from all participants. The blood samples, which were obtained by colorectal and pancreatic surgery teams, were randomly selected according to the stage of cancer (Table S2). All blood samples were collected in a blood collection tube treated with K_2_ EDTA, and plasma was transferred after centrifugation at a rate of 1,500 × *g* for 15 min (4 °C), according to protocol from the previous study^22^. We used a QIAamp Circulating Nucleic Acid Kit (55114, Qiagen, Hilden, Germany) for extraction of cell-free DNA from clinical specimens of three CRC patients. Approximately 100 μL cfDNA was extracted using 500 μL of each clinical sample. The extracted cfDNA was screened by real-time PCR and stored at − 20 °C until use.

### Cell-free DNA integrity and cellular DNA background from clinical specimens

To compare the clinical utility of the conventional assay and the biocomposite platform, cfDNA integrity and cellular DNA background from the clinical samples were measured using *Alu* element primers (247 and 115 bp products), and β-actin primer (400 bp product)^22^. The C_T_ values for each clinical specimen were converted to DNA concentration, and cfDNA integrity was measured at *Alu* 247/115 ratio. A ratio close to 1.0 indicated that the cfDNAs were not truncated, whereas that close to 0 indicated that cfDNAs were truncated^22^. The C_T_ values of β-actin present cellular DNA background. Extracted cfDNA contaminated with cellular DNA has a lower Ct value than uncontaminated cfDNA^22^.

## Results and discussion

### Design of the biocomposite platform

**Figure 1:**
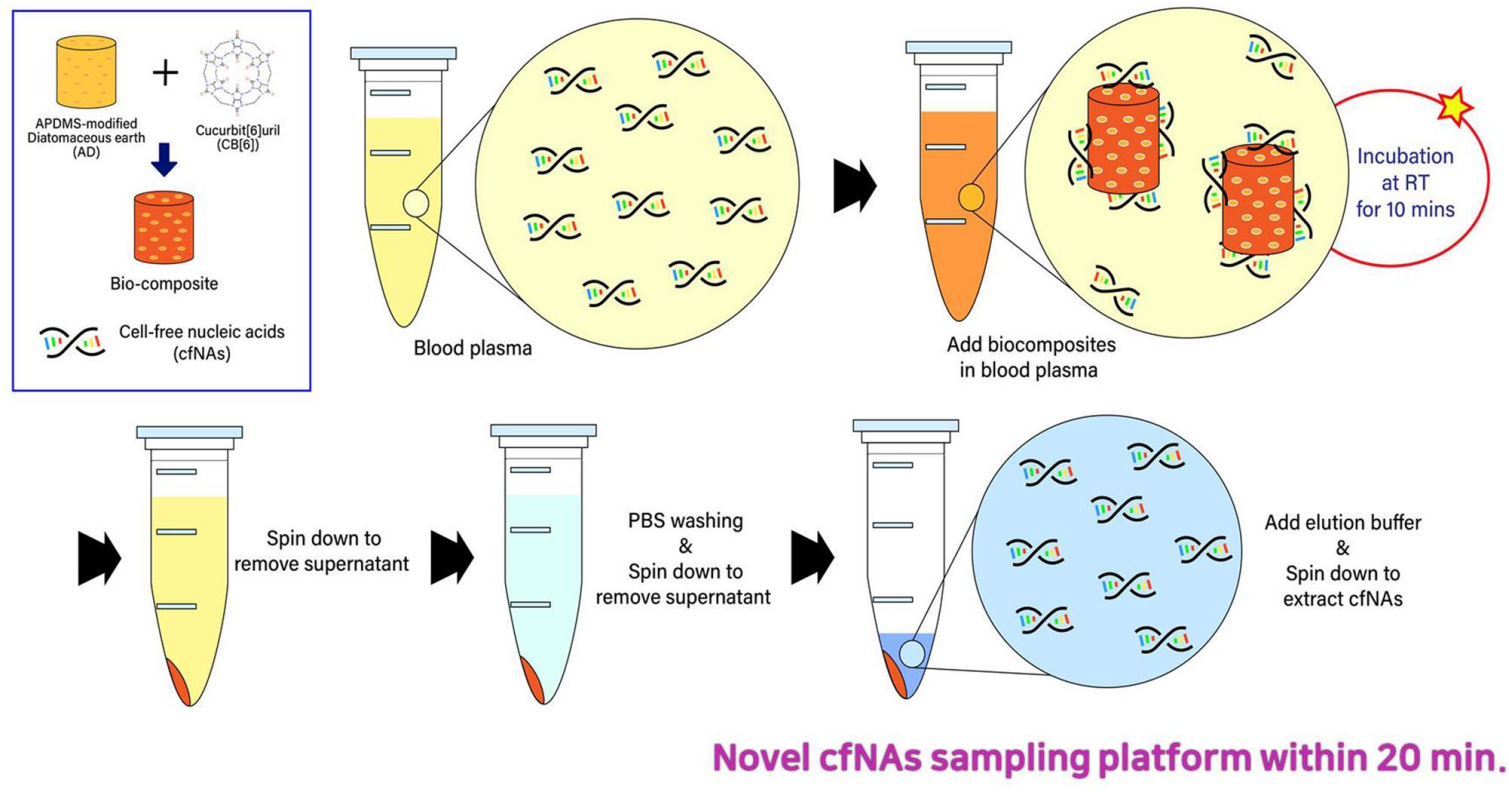
Schematic representation of the process of cfNA sampling from clinical specimens using a complex of amine-modified diatomaceous earth (DE) coated by cucurbit[6]uril (CB) (the biocomposite platform). First, the biocomposite platform was prepared for ready-to-use cfNA sampling. The biocomposites were added to blood plasma samples and incubated at RT for 10 min with shaking for 30 s at 2 min intervals for cfNAs capture. The solution was centrifuged to remove the supernatant containing debris, and the precipitate was washed three times with 1 mL PBS. Finally, high pH (pH 10.6) elution buffer was added and centrifuged to extract cfNAs. The biocomposite platform was able to isolate cfNAs from clinical specimens at high concentrations and purity within 20 min without requiring large equipment or a thermo-regulator.

Figure 1 is a schematic showing the construction of the biocomposite platform based on amine-modified DE coated with CB, and a description of the experimental procedure used to isolate cfNA from plasma specimens using the biocomposite platform. DE modified by 3-aminopropyl-methyl-diethoxysilane (APDMS) has amine groups on the outer and inner surface that render it chemically stable in aqueous solution. The positively charged amine-modified DE interacts with the negatively charged carbonyl portals of CB, resulting in an electrically neutralised and stabilised complex. In addition, we confirmed that the biocomposite have several micropores, and a large surface area (Fig. S2A). The biocomposite has a high molecular recognition capability by the porous structure of DE and the cavity structure of CB, and this function enhances the capture efficiency of the biocomposite for cfDNA isolation. Furthermore, we performed FTIR analysis to identify the properties of the biocomposite after the cfDNA binding. The wavelength peaks at 1681 cm^−1^ (C=O bonds) and 1,071 cm^−1^ (C–N bonds) were observed in the biocomposite group (Fig. S2B). The C=O bonds at the edge of CB reacts with NH_2_ of AD to act as one of the important anchoring site in the biocomposite platform^30^. In addition, the C–N bonds indicate that the oxygen atoms, which are located along the edges of CB, which can bind with the amine group from the AD. Therefore, these characteristics of AD and CB contribute to improve the structural stability of the biocomposite for cfDNA capturing. The biocomposite platform can be used to detect various biomarker molecules for sampling of clinical specimens. The application of the biocomposite platform includes the following steps (Fig. 1): (1) *Mixing of blood plasma with biocomposite* the biocomposite is mixed with blood plasma samples and shaken by hand to capture cfDNA. (2) *Binding to cfDNA* the amine groups of the fragmented cfDNA covalently bind to the C=O of the carbonyl portal of CB on the DE. The phosphate backbone of cfDNA can electrostatically bind to positively charged DE and CB in the biocomposite. (3) Washing to remove the debris: two binding mechanisms lead to a stable combination of the biocomposites and cfDNA. (4) Elution of isolated cfDNA: the covalent and electrostatic bonds between the biocomposite and cfDNA are cleaved using the elution buffer (pH 10.6), resulting in high efficiency isolation of cfDNA. The biocomposite platform enabled rapid sampling of cfDNA within 20 min, and the product obtained showed good quality and quantity.

### Synthesis and optimisation of biocomposites

**Figure 2:**
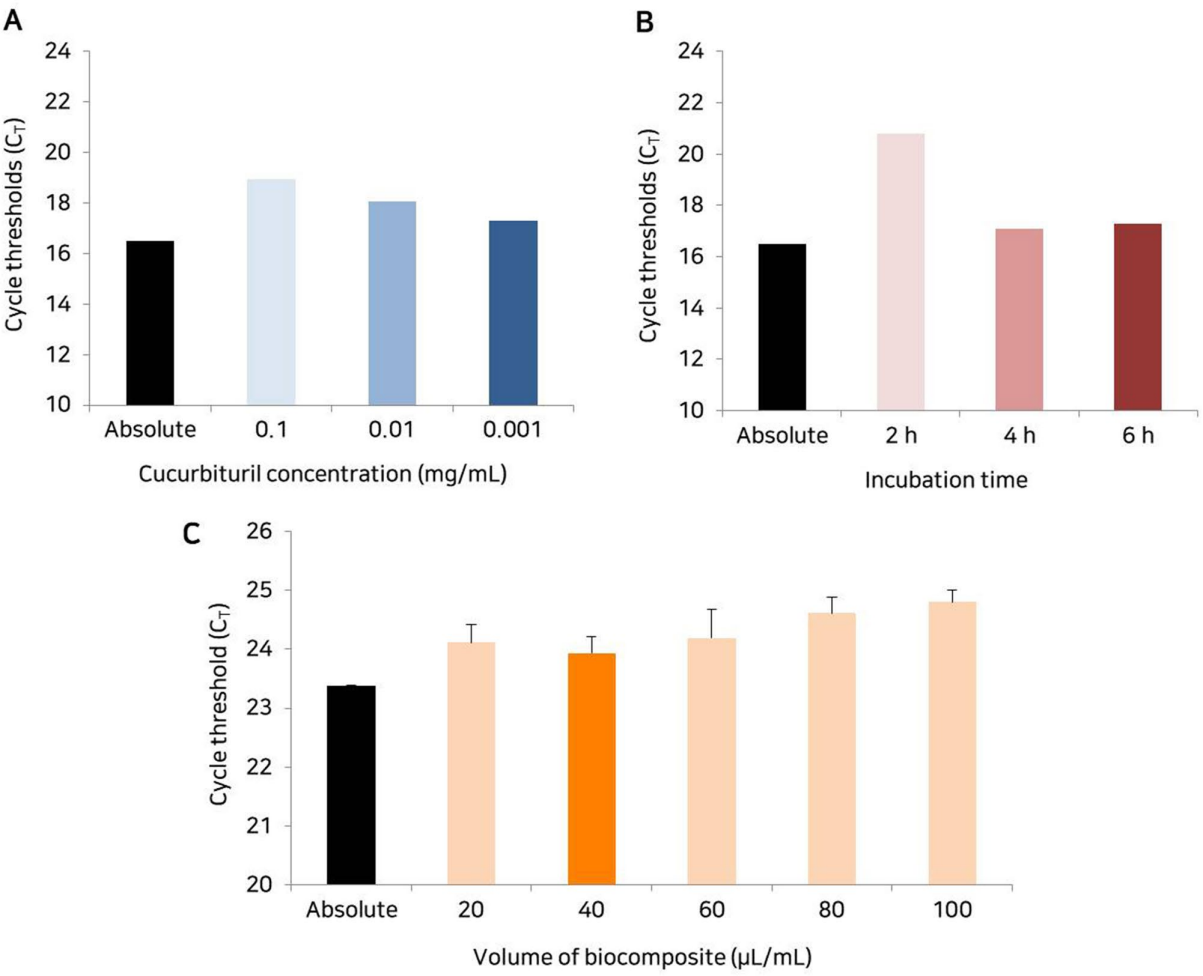
Optimisation of the biocomposite platform. (**A**) Analysis of capture efficiency using 1 ng of amplified 777 bp DNA according to CB concentration (0.1, 0.01, and 0.001 mg/mL) with 50 mg AD and 6 h of incubation time. The absolute (black) means 1 ng of amplified 777 bp DNA without CB. (**B**) Analysis of capture efficiency using 1 ng of amplified 777 bp DNA according to incubation time (2, 4, and 6 h) for the construction of the biocomposite platform with 50 mg AD and 0.001 mg/mL CB. (**C**) Analysis of capture efficiency using 10^4^ cellular gDNA according to the volume of biocomposite (20, 40, 60, 80, and 100 μL/mL) with 50 mg AD, 0.001 mg/mL CB, and 6 h of incubation time. Colours represent results of the absolute (black) and the biocomposite platform (blue, red, and orange). The absolute (black) means 10^4^ cellular gDNA without the biocomposite. Error bars indicate standard deviation from the mean based on at least three independent experiments.

A protocol for the synthesis of biocomposites was designed by optimising DE and CB concentrations, incubation time, and the volume of biocomposites to produce molecules with high functionality for cfDNA sampling. To optimise the protocol for the synthesis of biocomposites, human gDNA and an amplified 777 bp DNA product were prepared. Different CB concentrations (0.1, 0.01, and 0.001 mg/mL) and incubation times (2, 4, and 6 h) were tested to determine the optimal conditions for the generation of biocomposites. Determining the optimal concentration of CB for coating the DE is essential to improve the efficiency of cfDNA isolation (Fig. 2A). High capture efficiency for 1 ng of a 777 bp product was observed using a CB concentration of 0.001 mg/mL and 50 mg DE. The optimal incubation time for DE and CB binding is essential to obtain stabilised biocomposites (Fig. 2B). An incubation time of 4 h with 50 mg DE and 0.001 mg/mL CB showed high capture efficiency for 1 ng of a 777 bp product. Different volumes (20, 40, 60, 80, and 100 μL) of biocomposite were tested to identify the ideal volume yielding high quality and quantity cfDNA (Fig. 2C). The volume of the biocomposites is an important factor to capture high concentrations of cfDNA and reduce uncaptured cfDNA. The results showed that 40 μL of biocomposites resulted in high capture efficiency of gDNA from 10^4^ cells. These optimal conditions for the synthesis of biocomposites were used for further characterisation of the biocomposite platform using clinical specimens.

### Characterisation of the biocomposite platform

**Figure 3:**
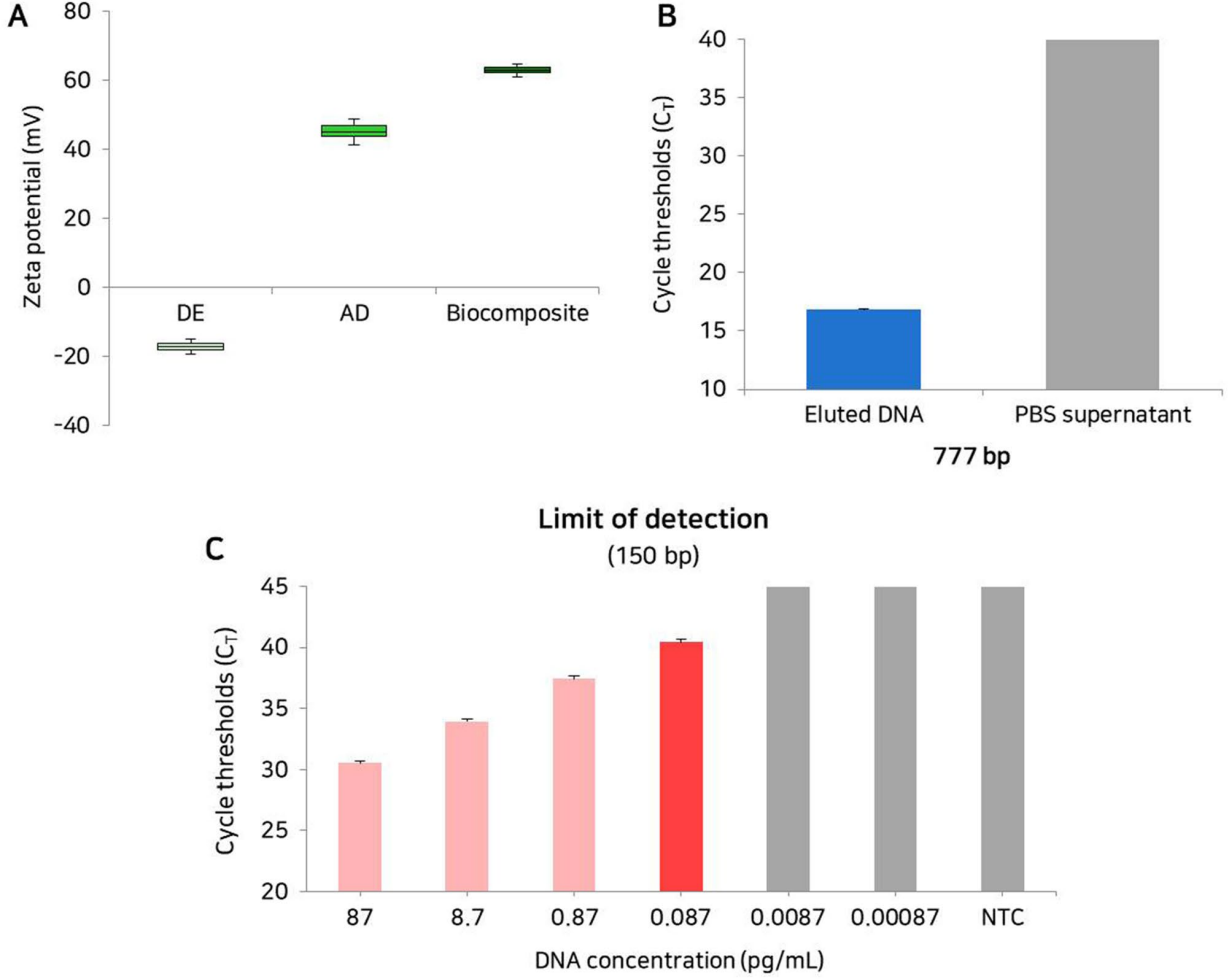
Characterisation of the biocomposite platform. (**A**) Box plot for the comparison of zeta potential between DE, AD, and the biocomposite. (**B**) Confirmation of the capture efficiency of DNA using biocomposite platform-eluted DNA and the PBS supernatant after the PBS washing step. (**C**) Limit of detection of the biocompostie platform. The bars represent the results from the biocomposite platform depends on the concentrations of targets (red) and negative control (grey). Error bars indicate standard deviation from the mean based on at least three independent experiments.

**Figure 4:**
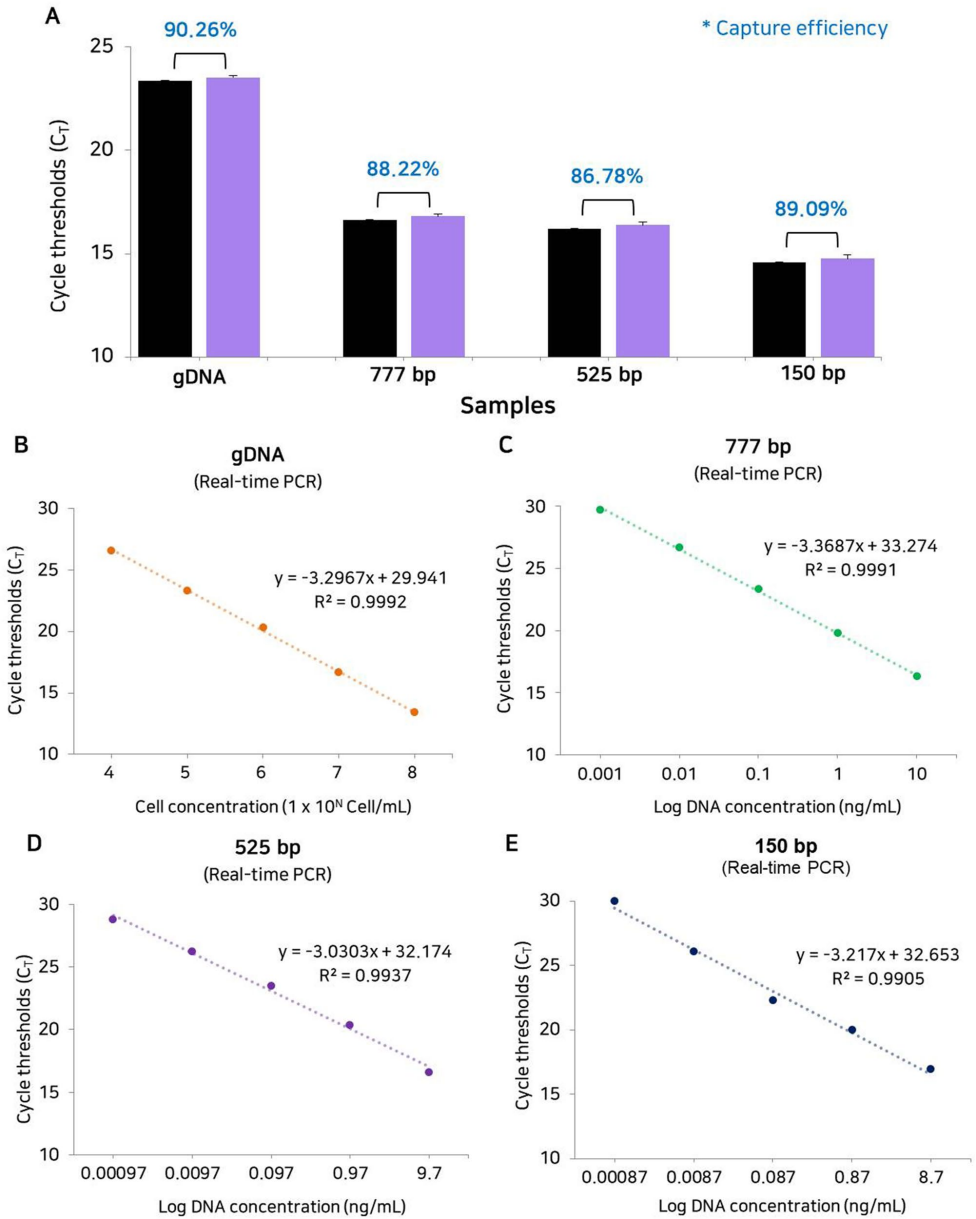
Capture efficiency of the biocomposite platform. (**A**) Measurement of the capture efficiency of the biocomposite platform using gDNA, amplified 777, 525, and 150 bp DNA. Colours represent results of input amount of target (black) and the biocomposite platform (violet). Error bars indicate standard deviation from the mean based on at least three independent experiments. (**B**) Detection of gDNA from HCT116 cells using a 102 bp primer. (**C**) Detection of amplified 777 bp DNA using a 133 bp primer. (**D**) Detection of amplified 525 bp DNA using a 203 bp primer. (**E**) Detection of amplified 150 bp DNA using a 150 bp primer.

The zeta potential of DE, amine-modified DE, and biocomposites was measured (Fig. 3A, Table S3). The zeta potential measures the surface charge of nanoparticles, which is an indicator of the stability of colloidal dispersion because it represents the resistance between adjacent charged particles^36^. Composites with a high negative or positive zeta potential have a high dispersion stability because of the lack of aggregation between particles, whereas a low zeta potential is associated with coagulation or flocculation caused by high attraction between particles^36^. The surface charge of the biocomposite was more positive than that of pure DE and AD, indicating that the biocomposites had excellent dispersion stability (more than + 61 mV) in solution (Fig. 3A, Table S3). Therefore, cfDNAs can be captured with the positive charge of the biocomposite by the strong electrostatic interaction. Next, we tested the capture efficiency of the biocomposite platform with gDNA and amplified 777, 525, and 150 bp products. The biocomposite platform showed high capture efficiency for the 777 bp amplified DNA product without DNA loss during the PBS wash (Fig. 3B). As the result of real-time PCR, the C_T_ value of the DNAs in the supernatant collected after the PBS wash were measured. The results showed that the optimised biocomposite platform could capture most of the drifting DNA with high probability and showed strong binding that was not disrupted during the PBS washing step. Next, we examined the sensitivity of the biocomposite platform with 150 bp DNA products (Fig. 3C). We used serially diluted 150 bp DNA products to confirm the sensitivity of the biocomposite platform. We confirmed that the detection limit of biocomposite platform was down to 8.7 fg/mL (converted to 5.37 × 10^2^ copies/mL). Furthermore, we examined the recovery rate of the biocomposite platform using a gDNA and fragmented DNAs (777, 525 and 150 bp), and the real ratio was calculated based on the standard curve of each sample (Fig. 4). The capture efficiency depends on the fragmented size of cfDNAs is important to confirm the prognosis information of clinical patients^37^. The four types of DNA obtained using the biocomposite platform showed similar C_T_ values before and after capture. The rate of capture according to input DNA (DNA concentration with capture/input DNA concentration × 100%) was calculated, and the capture efficiency was measured as 90.26%, 88.22%, 86.78%, and 89.09% for gDNA, 777, 525, and 150 bp DNA products, respectively (Table S4). These results indicate that the biocomposite platform is a simple and rapid sampling technique that can capture cfDNA with high efficiency.

### Clinical utility of the biocomposite platform

**Figure 5:**
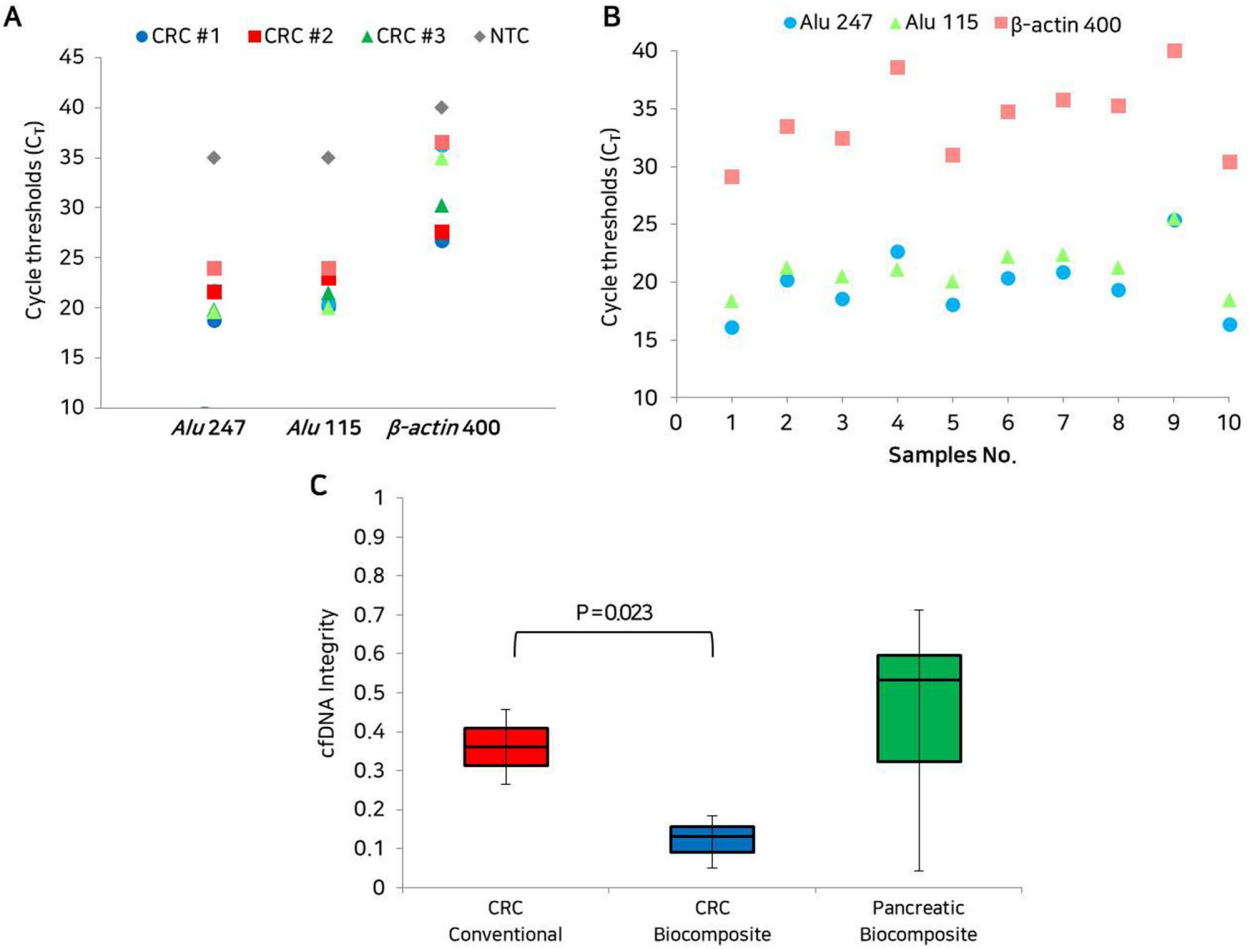
Clinical utility of the biocomposite platform. (**A**) Dot plots of the detection of *Alu* 247 bp, *Alu* 115 bp, and β-actin 400 bp using samples from three CRC patients. Circles represent CRC #1 (blue), squares represent CRC #2 (red), triangles represent CRC #3 (green), and diamonds represent negative control (NTC) samples (grey). Colours represent conventional assay (dark) and the biocomposite platform (light). (**B**) Dot plots of the detection of *Alu* 247 bp, *Alu* 115 bp and β-actin 400 bp using ten pancreatic cancer patient samples with the biocomposite platform. Light blue circles represent *Alu* 247 bp, light green triangles represent *Alu* 115 bp, and light red squares represent β-actin 400 bp in each sample. (**C**) Box plots of cfDNA integrity in three CRC and ten pancreatic cancer samples. Colours represent the conventional assay (red) in three CRC samples, the biocomposite platform (blue) in CRC samples, and the biocomposite platform (green) in pancreatic cancer samples. The p-value was evaluated by Student t test.

The clinical utility of the biocomposite platform for cfDNA sampling was tested using liquid biopsy samples from 13 cancer patients. First, plasma cfDNA obtained from three patients with CRC was analysed (Fig. 5A, Table S5). The cfDNAs isolated from the plasma were analysed by real-time PCR using *Alu* 247 bp, *Alu* 115 bp, and β-actin 400 bp gene fragments. The C_T_ data obtained by real-time PCR allow determination of the absolute amount of longer fragments of plasma DNA, the total amount of cfDNA in plasma, and the cellular DNA background based on the standard curve of each sample (Fig. S1). The integrity of cfDNA was then determined using the *Alu* 247/115 ratio, which was close to zero (0). This indicated that most of the DNA was truncated as cfDNA (Fig. 5C, Table S4). In CRC samples, the cfDNA integrity index determined using the biocomposite platform was lower (0.18) than that obtained using the conventional method (0.27). This result confirmed that the biocomposite platform easily captured the small DNA fragments, which can be regarded as reliable evidence of the whole tumor burden^38^. The cellular DNA background using the biocomposite platform, the DNAs were amplified with the β-actin 400 bp primer. The amplification efficiency of the biocomposite platform was higher (C_T_ value, 35.89) than that of the conventional method (C_T_ value, 28.20). This result confirmed that the biocomposite platform minimised the cellular background signal by eliminating the lysis step. In addition, the plasma cfDNA of ten pancreatic cancer patients was analysed (Fig. 5B, Table S5). The cfDNA integrity determined by the *Alu* 247/115 ratio was 0.15–0.71 (Fig. 5C, Table S6). The C_T_ value for the β-actin 400 bp gene was high (C_T_ value, 34.05) in the biocomposite platform. Taken together, the results indicate that the biocomposite platform is a useful method for capturing cfDNA from clinical specimens within 20 min, and that it can overcome the limitations of conventional approaches.

## Conclusions

A novel sampling platform for the simple and rapid isolation of cfDNA from liquid biopsy specimens from cancer patients with low background molecule concentrations and a high yield of cfDNA would be a useful tool for clinical use. An effective method would improve the early diagnosis of cancer and metastatic disease by detecting cancer-derived ctDNA, and it could help to predict treatment outcomes and patient prognosis^39,40^. Many ctDNA analysis, methods were developed in recent years to increase the accuracy, sensitivity, and specificity of cfDNA detection^13,41–43^. However, the existing methods are expensive, complex, and require high and low temperature conditions and chaotropic reagents for cell lysis. There is currently no established method for extracting cfNAs, and the analysis of cfNAs is therefore limited by low sensitivity and specificity because of the low amount and purity of cfNAs isolated. Recently, one study described for the standardized (pre)analytical work flow for cfDNA by multicenter based testing^44^. They have examined six commercialized kits for cfDNA isolation from spiked DNA in blood samples, not real samples. Although the study was given the insights regarding quantification, downstream analysis, process analysis, assay design and validation of cfDNA, the commercialized kits required several instruments for cfDNA isolation as well as there are expensive methods^44^.

In this study, we report the synthesis of a biocomposite of amine-modified DE and CB for sampling of cfNAs that overcomes the limitations of conventional methods for clinical use. This biocomposite platform is a simple, rapid, and cost-effective cfNA isolation system. The biocomposite platform has several advantages, as follows: first, the biocomposite platform does not require large equipment. Conventional methods require specialised equipment such as vacuum pumps, high-speed centrifuges, and temperature-regulated chambers for the lysis process. On the other hand, the biocomposite platform can be used for point-of-care testing using only a mini-centrifuge with a built-in battery. Second, the biocomposite platform (less 3$) is more economical method than spin column based assay (over 30$ with QIAamp Circulating Nucleic Acid Kit). Third, the biocomposite platform has high capture efficiency (86.78–90.26%), as demonstrated using three types of DNA. Fourth, the biocomposite platform can extract cfNAs with high purity in the clinical setting. There is no lysis step, which can decrease the cellular DNA background derived from normal cells. Fifth, the cfDNA isolated by the biocomposite platform showed a lower integrity ratio than that obtained by the conventional method. This method can extract high amounts of cfDNA containing lower amounts of longer DNA fragments, which contributes to the high correlation of cfNA analysis. Finally, the biocomposite platform can extract cfNAs quickly within 20 min. The biocomposite platform thus allows high efficiency cfNA sampling for early diagnosis and prognosis prediction in human cancers, including CRC and pancreatic cancer. Nevertheless, further study would be desired to optimize the protocol with a large clinical cohort for improving the sensitivity and specificity for the cfDNA isolation in clinical applications. This simple and low-cost cfNA sampling method based on the biocomposite platform could be useful for cancer diagnosis, monitoring, and determining treatment outcomes.

## Supplementary information

Supplementary Information

## Abbreviations

cfNA: Cell-free nucleic acid
DE: Diatomaceous earth
CB: Cucurbituril
ctDNA: Circulating tumour deoxyribonucleic acid
cfDNA: Cell-free deoxyribonucleic acids
CRC: Colorectal cancer
APDMS: 3-Aminopropyl-methyl-diethoxysilane
DW: Distilled water
RT: Room temperature
dNTP: Deoxynucleotide triphosphate
BRC: Bio resource center
IBR: Institutional review board
C_T_: Cycle threshold
