# Supplementary material for: A biocomposite-based rapid sampling assay for circulating cell-free DNA in liquid biopsy samples from human cancers
Source: Sci Rep. 2020 Sep 10;10:14932. doi: 10.1038/s41598-020-72163-8 (PMC7484795; doi:10.1038/s41598-020-72163-8)
Supplement: Supplementary file 1 — Supplementary Information [file 41598_2020_72163_MOESM1_ESM.docx]

**Supplementary**

**A biocomposite-based rapid sampling assay for circulating cell-free DNA in liquid biopsy samples from human cancers**

Bonhan Koo^1,2,+^, Eunsung Jun^1,4,+^, Huifang Liu^1,2^, Eo Jin Kim^1^, Yun-Yong Park^1^, Seok-Byung Lim^3^, Song Cheol Kim^2,4,*^, Yong Shin^1,2,*^

^1^Department of Convergence Medicine, Asan Medical Institute of Convergence Science and Technology, Asan Medical Center, University of Ulsan College of Medicine, Seoul, Republic of Korea

^2^Biomedical Engineering Research Center, Asan Institute of Life Science, Asan Medical Center, Seoul, Republic of Korea

^3^Division of Colon and Rectal Surgery, Department of Surgery, Asan Medical Center, University of Ulsan College of Medicine, Seoul, Republic of Korea

^4^Division of Hepatobiliary and Pancreatic Surgery, Department of Surgery, Asan Medical Center, University of Ulsan College of Medicine, Seoul, Republic of Korea

^+^ These authors contributed equally to this work.

*To whom correspondence should be addressed. Email: [shinyongno1@gmail.com](mailto:shinyongno1@gmail.com) & [drksc@amc.seoul.kr](mailto:drksc@amc.seoul.kr)

**
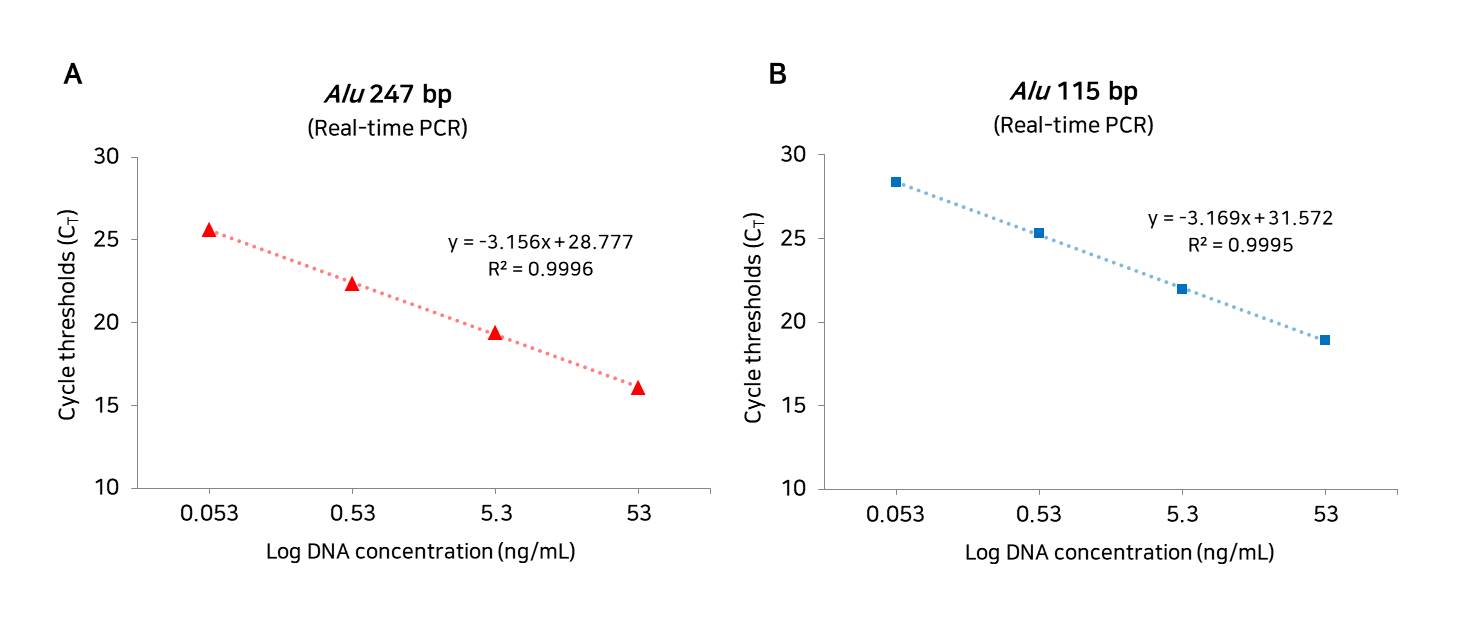
Figure S1.** Linear relationship between sample concentration and C_T_ values of the fluorescence signal determined by real-time PCR. (A) Detection of longer fragments of plasma DNA from healthy controls using the *Alu* 247 bp primer. (B) Detection of the total amount of cfDNA from healthy controls using the *Alu* 115 bp primer.


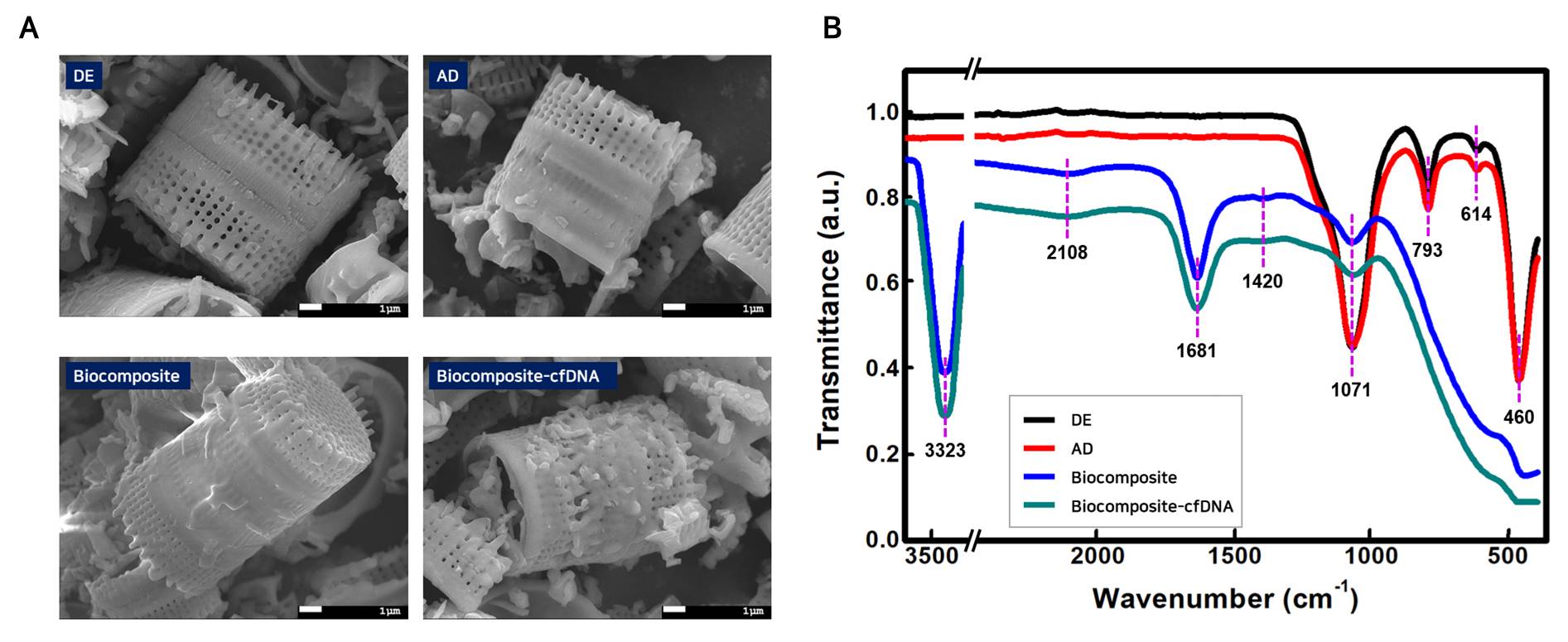


**Figure S2.** Characterization of material structure in biocomposite platform. (A) Scanning electron microscopy (SEM) images of the DE, AD, biocomposite, and biocomposite-cfDNA. (B) Visual Fourier transform infrared spectroscopy (FTIR) spectrum analysis of the DE, AD, biocomposite, and biocomposite-cfDNA.

**Table S1.** Primer sequences used for the conventional PCR assay.

| Target gene | | Sequence (5' → 3') |
| --- | --- | --- |
| *β-actin* | 102 bp F | ATC GCC GAC AGG ATG CA |
|  | 102 bp R | CGT ACT CCT GCT TGC TGA TCC |
|  | 400 bp F | GCA CCA CAC CTT CTA CAA TGA |
|  | 400 bp R | TGT CAC GCA CGA TTT CCC |
| *KRAS* | 133 bp F | GCT GTA TCG TCA AGG CAC TCT T |
|  | 133 bp R | ACC TTA TGT GTG ACA TGT TCT AAT ATA GTC |
|  | 150 bp F | GCA GAA CAG CAG TCT GGC TAT TT |
|  | 150 bp R | CAG TTG ACT GCA GAC GTG TAT CG |
|  | 777 bp F | TAG CCG CCG CAG AAC AGC AGT C |
|  | 777 bp R | TCA CAA TAC CAA GAA ACC CAT AAA |
| *C. burnetii*  *IS1111a* | 203 bp F | GAG CGA ACC ATT GGT ATC G |
|  | 203 bp R | CTT TAA CAG CGC TTG AAC GT |
|  | 525 bp F | CGG GTT AAG CGT GCT CAG TAT GTA |
|  | 525 bp R | TGC CAC CGC TTT TAA TTC CTC CTC |
| *Alu segment* | 247 bp F | GTG GCT CAC GCC TGT AAT C |
|  | 247 bp R | CAG GCT GGA GTG CAG TGG |
|  | 115 bp F | CCT GAG GTC AGG AGT TCG AG |
|  | 115 bp R | CCC GAG TAG CTG GGA TTA CA |

**Table S2.** Clinical characteristics of three patients with colorectal cancer and ten patients with pancreatic cancer.

|  | Nr. | Age | | Gender | | Pathologic  Stage |
| --- | --- | --- | --- | --- | --- | --- |
| Colorectal  cancer | CRC #1 | 64 | F | | 2 | |
|  | CRC #2 | 42 | M | | 3 | |
|  | CRC #3 | 72 | F | | 3 | |
| Pancreatic  cancer | P #1 | 71 | F | | 2 | |
|  | P #2 | 59 | M | | 3 | |
|  | P #3 | 70 | M | | 3 | |
|  | P #4 | 64 | F | | 2 | |
|  | P #5 | 47 | M | | 3 | |
|  | P #6 | 54 | M | | 2 | |
|  | P #7 | 54 | M | | 1 | |
|  | P #8 | 56 | F | | 2 | |
|  | P #9 | 72 | F | | 1 | |
|  | P #10 | 53 | M | | 1 | |

**Table S3.** Comparison of the zeta potential between DE, AD, and the biocomposite.

| Zeta potential  (mV) | Test #1 | Test #2 | Test #3 | Test #4 | Test #5 | Test #6 | **Test mean** |
| --- | --- | --- | --- | --- | --- | --- | --- |
| DE | -15.47 | -14.23 | -13.94 | -16.36 | -16.53 | -14.89 | **-15.24** |
| AD | 46.73 | 47.48 | 44.04 | 43.09 | 47.37 | 44.04 | **45.46** |
| Biocomposite | 64.24 | 62.08 | 62.55 | 61.92 | 63.58 | 63.81 | **63.03** |

**Table S4.** Capture efficiency determined using gDNA, amplified 777, 525, and 150 bp DNA in the biocomposite platform.

|  | Test #1  (C_T_) | Test #2  (C_T_) | Test #3  (C_T_) | Test mean  (C_T_) | Absolute  (C_T_) | **Capture efficiency**  **(%)** |
| --- | --- | --- | --- | --- | --- | --- |
| gDNA | 23.63 | 23.51 | 23.44 | 23.53 | 23.38 | **90.26** |
| 777 bp | 16.81 | 16.94 | 16.72 | 16.82 | 16.64 | **88.22** |
| 525 bp | 16.56 | 16.34 | 16.29 | 16.40 | 16.21 | **86.78** |
| 150 bp | 14.68 | 14.96 | 14.64 | 14.76 | 14.59 | **89.09** |

**Table S5.** Comparison of the C_T_ values of *Alu* 247 bp, *Alu* 115 bp, and β-actin 400 bp from three colorectal cancer specimens using the conventional assay and biocomposite platform, and from ten pancreatic cancer specimens using the biocomposite platform.

|  | Nr. | ***Alu* 247 bp** | | ***Alu* 115 bp** | | ***β-actin* 400 bp** | |
| --- | --- | --- | --- | --- | --- | --- | --- |
|  |  | Conventional  (C_T_) | Biocomposite  (C_T_) | Conventional  (C_T_) | Biocomposite  (C_T_) | Conventional  (C_T_) | Biocomposite  (C_T_) |
| Colorectal  cancer | CRC #1 | 18.76 | 20.11 | 20.11 | 20.39 | 26.69 | 36.19 |
|  | CRC #2 | 21.63 | 22.97 | 22.57 | 22.93 | 27.63 | 36.5 |
|  | CRC #3 | 19.83 | 21.51 | 21.51 | 20.02 | 30.29 | 34.98 |
| Pancreatic  cancer | P #1 | . | 16.09 | . | 18.37 | . | 29.11 |
|  | P #2 | . | 20.19 | . | 21.26 | . | 33.43 |
|  | P #3 | . | 18.55 | . | 20.49 | . | 32.42 |
|  | P #4 | . | 22.66 | . | 21.08 | . | 38.53 |
|  | P #5 | . | 18.05 | . | 20.11 | . | 31.00 |
|  | P #6 | . | 20.38 | . | 22.22 | . | 34.70 |
|  | P #7 | . | 20.87 | . | 22.41 | . | 35.77 |
|  | P #8 | . | 19.32 | . | 21.30 | . | 35.25 |
|  | P #9 | . | 25.34 | . | 25.55 | . | 40.00 |
|  | P #10 | . | 16.33 | . | 18.46 | . | 30.36 |

**Table S6.** Comparison of cfDNA integrity between the conventional assay and the biocomposite platform in three colorectal cancer specimens and the biocomposite platform in ten pancreatic cancer specimens.

|  | Nr. | **cfDNA integrity index**  (0 ≤ X ≤ 1) | |
| --- | --- | --- | --- |
|  |  | Conventional | Biocomposite |
| Colorectal  cancer | CRC #1 | 0.36 | 0.05 |
|  | CRC #2 | 0.27 | 0.13 |
|  | CRC #3 | 0.46 | 0.18 |
| Pancreatic  cancer | P #1 | . | 0.71 |
|  | P #2 | . | 0.29 |
|  | P #3 | . | 0.55 |
|  | P #4 | . | 0.04 |
|  | P #5 | . | 0.60 |
|  | P #6 | . | 0.51 |
|  | P #7 | . | 0.41 |
|  | P #8 | . | 0.57 |
|  | P #9 | . | 0.15 |
|  | P #10 | . | 0.64 |
